# Wake-up intracerebral hemorrhage: hematoma expansion and outcomes

**DOI:** 10.3389/fneur.2025.1620170

**Published:** 2025-07-25

**Authors:** Tove Almqvist, Anna Falk Delgado, Christina Sjöstrand, Michael V. Mazya

**Affiliations:** ^1^Department of Clinical Neuroscience, Karolinska Institutet, Stockholm, Sweden; ^2^Department of Neurology, Karolinska University Hospital, Stockholm, Sweden; ^3^Department of Neuroradiology, Karolinska University Hospital, Stockholm, Sweden; ^4^Department of Neurology, Danderyd Hospital, Stockholm, Sweden

**Keywords:** intracerebral hemorrhage, acute stroke, computed tomography, mortality, outcomes assessment

## Abstract

**Introduction:**

While understudied, wake-up intracerebral hemorrhage (WU-ICH) is not uncommon (8.8–20.3% of ICH patients). Since the risk of hematoma expansion (HE) decreases as time passes, an uncertain onset time in WU-ICH may influence the risk of in-hospital HE and the potential effects of HE-preventive treatments. We aimed to evaluate HE and outcomes in WU-ICH compared to known-onset ICH.

**Methods:**

We included ICH patients admitted to the Karolinska University Hospital from 2016 to 2022, comparing WU-ICH vs. known-onset ICH regarding baseline characteristics, HE, and outcomes.

**Results:**

Of 763 patients, 147 (19%) had WU-ICH and 616 (81%) had known onset, median (IQR) last-known-well to hospital time 9.6 h (5.9–12.2 h) vs. 1.3 h (0.9–2.0 h). WU-ICH patients more often had dementia (15% vs. 5%, *p* < 0.001), oral anticoagulants (26% vs. 16%, *p* = 0.005), and pre-stroke modified Rankin Scale 3–5 (24% vs. 15%, *p* = 0.01). Baseline ICH volume was 14 mL (6–35 mL) vs. 13 mL (5–34 mL). Among patients who underwent CT angiography at admission, 15% of WU-ICH vs. 27% of known-onset ICH had spot signs (*p* = 0.002). Of patients with CT follow-up <72 h, HE occurred in 24/77 (31.2%) in WU-ICH, and 123/356 (34.6%) in known-onset ICH, *p* = 0.57. Wake-up onset was not associated with HE in multivariable analysis, adjusted OR = 0.79 (95% CI 0.43–1.42). Analysis of the 3-month modified Rankin Scale showed no differences (median 4 vs. 4), unadjusted *p* = 0.35 and adjusted *p* = 0.78.

**Conclusion:**

WU-ICH had a similar risk of HE and similar 3-month outcomes as known-onset ICH. Excluding WU-ICH from future trials targeting HE may be unwarranted.

## 1 Introduction

Waking up with symptoms is common in intracerebral hemorrhage (ICH), ranging from 8.8 to 20.3%, although it is less frequent than in ischemic stroke (1–4). Studies comparing wake-up ICH (WU-ICH) and known onset ICH have reported varied results regarding differences in baseline and outcomes (1, 3, 4). Trials of therapies for ICH have not reported outcomes in the WU-ICH subgroup. Blood pressure lowering and hemostatic treatment aimed at preventing hematoma expansion (HE) work better in an early time window (5, 6). Because of the uncertainty of true onset time in WU-ICH, the risk of HE and the potential effects of treatments are difficult to estimate.

We aimed to evaluate whether the risk of HE in WU-ICH differs from known-onset ICH. Second, we aimed to establish whether 3-month functional outcomes and mortality in WU-ICH differ from those of known-onset ICH.

## 2 Materials and methods

### 2.1 Study population

Between 2016 and 2022, we included patients diagnosed with intracerebral hemorrhage (ICD-10 code I61) admitted to the Karolinska University Hospital, either as the first hospital or as a referral from another emergency hospital within the Stockholm Region. Patients were grouped into those who woke up with symptoms and those with a known symptom onset while awake. Patients with uncertain onset time who did not wake up with symptoms were excluded. Wake-up onset was defined as patients or next of kin stating that they experienced symptoms upon awakening, or patients found in bed during morning hours, with someone stating that they were well the day before. All etiologies of non-traumatic ICH were allowed except hemorrhagic transformation of ischemic stroke. All patients were treated according to local guidelines.

For evaluating HE, we included only patients who had a follow-up CT within 72 h after the first CT. Patients undergoing neurosurgery before a follow-up CT were excluded. For evaluating clinical characteristics, functional outcome, and mortality at 3 months, patients were included regardless of follow-up CT availability.

### 2.2 Ethics and data availability

Swedish Ethical Review Authority approval was obtained and the need for consent was waived due to the retrospective nature of the study. Anonymized data can be made available upon reasonable request from a qualified researcher.

### 2.3 Data collection and definitions

Data were collected from the local stroke quality registry using the Riksstroke platform and electronic health records. For examination of radiological scans, we used the Karolinska Sectra Picture Archiving and Communication System (PACS). This study followed the Strengthening the Reporting of Observational Studies in Epidemiology (STROBE) guidelines (7).

#### 2.3.1 Radiological variables

For our main analysis, HE was defined as expansion of total ICH volume by >6 mL or >33% or an increase in intraventricular hemorrhage (IVH) volume by >2 mL. Reporting IVH growth has been previously recommended (8, 9). For ICH < 1.5 mL, volume growth still needed to exceed 0.5 mL to be defined as HE. For sensitivity analyses, we used two common definitions of HE: >6 mL or >33%, and >12.5 mL or >33% (9). Volume was calculated semi-automatically using the volume tool in Sectra PACS. With this tool, the user draws a line between the two margins of the hematoma, and the software then identifies the hematoma and calculates its volume. Additional lines can be used to include additional hematoma components if needed. When the semiautomated measuring was considered inaccurate, that is, it could not separate ICH volume from IVH volume, we calculated total volume (ICH + IVH) using the semiautomated method and used the Kothari method (ABC/2) to calculate ICH volume (10). If several follow-up CTs were available within the first 72 h, we analyzed the CT closest to 24 h from the first one. For non-contrast CT (NCCT) markers, we used the definitions provided by Morotti et al. (9). Spot signs were analyzed in cases with available admission CTA, and defined as: “≥1 focus of contrast pooling within the ICH, with an attenuation ≥120 Hounsfield units (HU), discontinuous from normal or abnormal vasculature adjacent to the ICH, and being of any size and morphology” (11). We further collected data on widening of the temporal horns as a sign of hydrocephalus, concurrent SAH, and midline shift >4 mm. CT scans were evaluated by author TA (MD, doctoral student) after training and consensus reading with an experienced neuroradiologist (author AD).

#### 2.3.2 Clinical variables

Glasgow Coma Scale (GCS) scores, when missing, were approximated from electronic health records. NIH Stroke Scale (NIHSS) scores were only included when available. Modified Rankin Scale (mRS) scores at 3 months were based on electronic health records and Riksstroke’s 3-month follow-up questionnaire. Probable etiology was based on the structural lesion, medication, amyloid angiopathy, systemic/other disease, hypertension, undetermined (SMASH-U) system, with one modification: patients with ongoing oral anticoagulation (OAC) were not classified as having an ICH caused by medication (12). Instead, other etiologies were considered, such as hypertensive microangiopathy and cerebral amyloid angiopathy (CAA). OACs were instead seen as a contributing factor. Targeted blood pressure therapy was defined as initiating blood pressure-lowering treatment with a local guideline-recommended or individualized target level before surgery and within 24 h from onset, or keeping blood pressure within a target range if the baseline value was already in that range on arrival. Early neurological deterioration was defined as worsening of ≥ 2 GCS or ≥4 NIHSS points during the first 72 h. Last known well (LKW) for patients with wake-up onset was assumed to be 11 pm the day before onset if not clearly stated or impossible to extrapolate to another timepoint from information in electronic health records, since this has been reported to be the most common bedtime for individuals aged 45–70 (13). Onset times for patients with known onset were extracted from electronic health records. Pre-stroke dependence was defined as pre-stroke mRS 3–5.

### 2.4 Statistics

For univariate comparisons, the Mann–Whitney *U*-test, chi-square, and Fisher’s exact tests were used as appropriate. We considered *p* < 0.05 significant. We performed binary logistic regression on HE with multivariable adjustment to reduce potential bias from baseline imbalances. Inclusion of variables in the regression analysis was based on pre-determined clinical significance and baseline differences with a univariate *p* < 0.10. Wake-up onset was kept in the model throughout. Pre-determined variables were ICH volume at baseline, systolic blood pressure, infratentorial ICH, GCS, and antithrombotic use. Variance inflation factor was used to evaluate collinearity. Non-significant values (*p* > 0.05) were removed from the model. To evaluate the robustness of our results, we performed separate analyses with two common definitions for HE: (1) >6 mL or >33% and (2) >12.5 mL or >33%. These analyses were performed using the same method as in the main analysis. For variables with >10% missing values, we considered whether the variables were missing at random. For variables not missing at random, we performed sensitivity analyses. Additionally, we performed a sensitivity analysis using a composite endpoint of early neurological deterioration or death within 72 h. Details of this analysis can be found in the Supplementary material.

For comparison of 3-month mRS scores between wake-up and known-onset ICH, we performed univariable and multivariable ordinal regression, with results reported as adjusted common odds ratios for higher mRS scores. Variables were included in the same way as for HE. Here, we included age, pre-stroke dependence, dementia, and HE as pre-determined variables. The Brant test was used to test the proportional odds assumption. If violations in the model would occur in the univariable regression model, we would compare all possible dichotomized versions of the mRS: 0 vs. 1–6, 0–1 vs. 2–6, 0–2 vs. 3–6, etc. In the multivariable regression analysis, variables that violated the proportional odds assumption were removed. All data analyses were performed in R Studio.

## 3 Results

**Figure 1:**
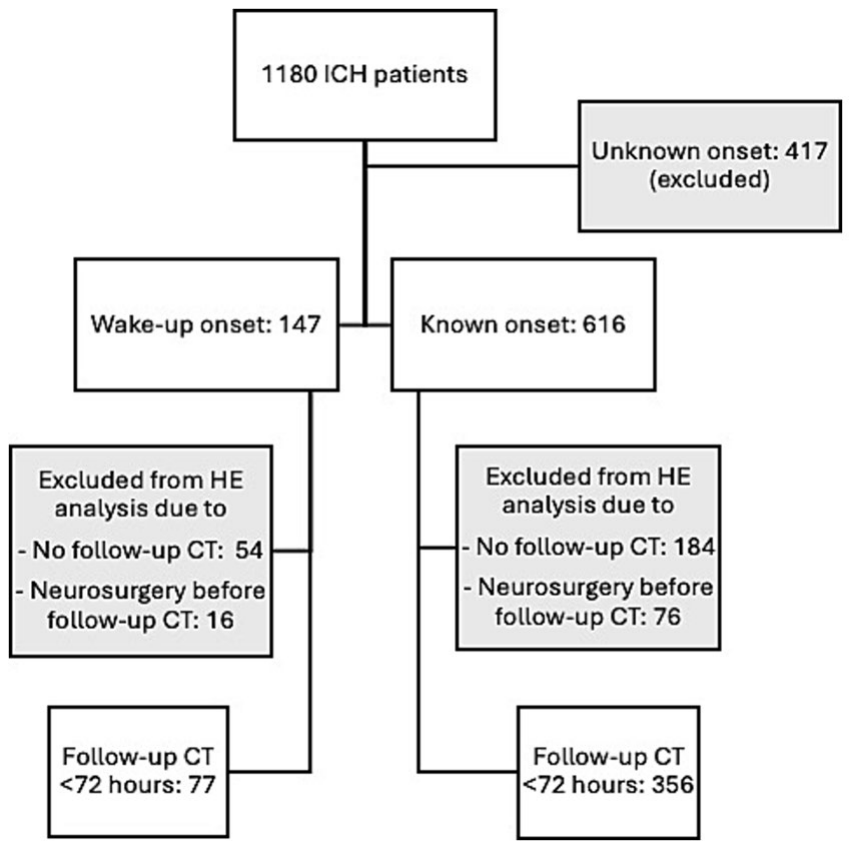
Flow chart of patient inclusion. CT, computed tomography; HE, hematoma expansion; ICH, intracerebral hemorrhage.

Of 1,180 ICH patients treated during the study period, the dataset for the present study was comprised of 147 (12.5%) cases with wake-up onset and 616 (52.2%) with a known onset (Figure 1). A total of 417 (35.3%) cases had an unknown, presumed awake onset; these were excluded from the analysis. Of the 763 cases in the study, 92 (12%) underwent neurosurgery before any follow-up CT was done. These could not be included in analyses of HE. Of the remaining 671 cases, CT follow-up <72 h was conducted in 433 (64.5%) patients.

Patients with WU onset and known onset were similar in age and sex, while dementia, oral anticoagulant and antihypertensive medication, atrial fibrillation, and pre-stroke dependence were more common in WU onset (Table 1). Median time from LKW to hospital arrival was 9.6 h (5.9–12.2 h) for WU-ICH, compared to 1.3 h (0.9–2.0 h) for known-onset ICH, *p* < 0.001. Symptom severity was similar, with median NIHSS 12 vs. 13 and median GCS 14 in both groups (Table 2).

**Table 1:** Demographics and medical history.

	WU ICH, *n* = 147		Known-onset ICH, *n* = 616		*p*
	*n*	% or median	*n*	% or median	
Age	147	71 (57–80)	616	67 (56–78)	0.09
Sex (f)	54/147	36.7%	259/6165	42.0%	0.24
Pre-stroke dependence	35/147	23.8%	93/615	15.1%	0.01
Living alone	43/145	29.7%	164/605	27.1%	0.54
Time from LKW to hospital arrival	141	9.6 h (5.9–12.2)	593	1.3 h (0.9–2.0)	<0.001
Medical history					
Previous ICH	9/147	6.1%	38/616	6.2%	0.98
Previous IS	19/147	12.9%	55/616	8.9%	0.15
Hypertension	90/147	61.2%	332/616	54.2%	0.12
Diabetes	24/147	16.3%	82/616	13.3%	0.35
Atrial fibrillation	40/147	27.2%	100/616	16.2%	0.002
Dementia or cognitive impairment	22/147	15.0%	32/616	5.2%	<0.001
Medications at onset					
Oral anticoagulant	38/147	25.9%	98/616	15.9%	0.005
Antiplatelet	31/147	21.1%	116/616	18.8%	0.53
Antihypertensive	85/147	57.8%	297/616	48.3%	0.04

ICH: intracerebral hemorrhage; IS: ischemic stroke; LKW: last known well; WU: wake-up.

**Table 2:** Clinical characteristics and acute treatment.

	WU ICH, *n* = 147		Known-onset ICH, *n* = 616		*p*
	*n*	% or median	*N*	% or median	
At admission					
NIHSS	98	12 (6–17)	411	13 (6–18)	0.85
GCS	147	14 (9–15)	615	14 (10–15)	0.57
Systolic blood pressure	141	170 (150–197)	591	172 (150–200)	0.33
ICH score	146	1.5 (1–3)	603	1 (1–2)	0.26
Etiology					
Hypertensive angiopathy	89/147	60.5%	348/616	56.5%	0.37
Amyloid angiopathy	17/147	11.6%	74/616	12.0%	0.88
Other	27/147	18.4%	113/616	18.3%	0.99
Uncertain	14/147	9.5%	81/616	13.1%	0.23
Medical treatments					
PCC	28/147	19.0%	78/609	12.8%	0.051
Vitamin K	15/146	10.3%	53/609	8.7%	0.55
Idarucizumab	0/147	-	1/609	0.2%	0.62
Targeted blood pressure treatment (if admission SBP > 140)	83/114	72.8%	376/480	78.3%	0.21
Neurosurgery	25/147	17.0%	136/616	22.1%	0.18

GCS: Glasgow Coma Scale; NIHSS: NIH Stroke Scale; ICH: intracerebral hemorrhage; IS: ischemic stroke; PCC: prothrombin complex concentrate; SBP: systolic blood pressure; WU: wake-up.

Median LKW to first CT scan was 10.3 h (6.7–14.0 h) in WU-ICH vs. 1.8 h (1.3–2.9 h) in known-onset ICH, *p* < 0.001 (Table 3). For patients with WU onset, CTA on arrival was done in 76/147 (51.7%), compared to 421/616 (68.3%) for known onset. Spot signs were seen in 22/76 (15%) vs. 169/421 (27.4%), *p* = 0.002. Multiple spot signs also occurred less frequently in WU-ICH, 5/76 (6.6%) vs. 79/412 (18.8%), *p* = 0.03. NCCT risk factors for HE were similarly frequent in WU-ICH and known-onset ICH (Table 3).

**Table 3:** Radiological findings and early outcomes.

	WU ICH, n = 147		Known-onset ICH, n = 616		*p*
	*n*	% or median	*n*	% or median	
Time from LKW to first CT	141	10.3 h (6.7–14.0)	593	1.8 h (1.3–2.9)	<0.001
Time from arrival to CT	141	37 min (21–63)	593	30 min (17–50)	0.004
First CT scan					
ICH volume (mL)	146	13.6 (5.5–35.0)	606	13.4 (5.0–33.7)	0.96
IVH	69/147	46.9%	282/616	45.9%	0.81
Hydrocephalus	59/147	40.1%	228/616	37%	0.48
Midline shift (>4 mm)	48/147	32.7%	195/616	31.8%	0.84
Blend sign	18/147	12.3%	59/616	9.8%	0.36
Black hole sign	41/147	28.1%	165/616	27.4%	0.86
Fluid sign	5/147	3.4%	14/616	2.3%	0.45
Barras scale density III–V	10/146	6.8%	38/603	6.3%	0.81
Barras scale shape III–V	13/146	8.9%	74/603	12.3%	0.25
ICH location					
Infratentorial	28/147	19.0%	93/616	15.1%	0.24
Deep hemispheric	73/147	49.7%	308/616	50.0%	0.94
Lobar hemispheric	42/147	28.6%	197/616	32.0%	0.42
Multiple locations	3/147	2.0%	5/616	8.1%	0.19
CTA on arrival,	76/147	51.7%	421/616	68.3%	<0.001
Spot sign(s)	22/76	15.0%	169/421	27.4%	0.002
Multiple spot signs	5/76	6.6%	79/421	18.8%	0.01
Neurological deterioration					
0–24 h	18/147	12.2%	120/616	19.5%	0.04
24–72 h	14/147	9.5%	38/616	6.2%	0.15
Follow-up CT scan	77/147	52.4%	356/616	57.8%	0.23
Time from first to follow-up CT	73	15.8 h (6.3–29.1)	342	12 h (5.8–22.0)	0.04
Hematoma expansion	24/77	31.2%	123/356	34.6%	0.57
Expansion (mL)	24	6.7 (1.9–12.0)	123	10.4 (2.9–20.6)	0.10
Expansion (%)	24	38.1% (13.5–76.3)	123	52.7% (22.4–121.6)	0.48

CT: computed tomography; CTA: computed tomography angiography; ICH: intracerebral hemorrhage; IVH: intraventricular hemorrhage; LKW: last know well; WU: wake-up.

Median time from initial CT to follow-up CT was 15.8 h (6.3–29.1) for WU-ICH and 12 h (5.8–22.0) for known-onset ICH, *p* = 0.04, excluding 92 cases that underwent neurosurgery before the first imaging follow-up and 238 patients who were not followed with a CT < 72 h based on clinical decision. In patients with CT follow-up <72 h, HE occurred in 24/77 (31.2%) in WU-ICH vs. 123/356 (34.6%) in known-onset ICH, *p* = 0.57 (Table 3). Neurological deterioration <24 h occurred in 18/147 (12.2%) in WU-ICH vs. 120/616 (19.5%) in known-onset ICH, *p* = 0.04.

In sensitivity analysis comparing known risk factors for HE in cases with vs. without follow-up CT, no significant differences were found. Mortality at 3 months was, however, higher in patients without a follow-up CT, 135/322 (42%) vs. 108/408 (26%), *p* < 0.001. Excellent outcome (mRS 0–1) was more common in the group without a follow-up CT, 43/322 (13%) vs. 33/408 (8%), *p* = 0.02.

**Figure 2:**
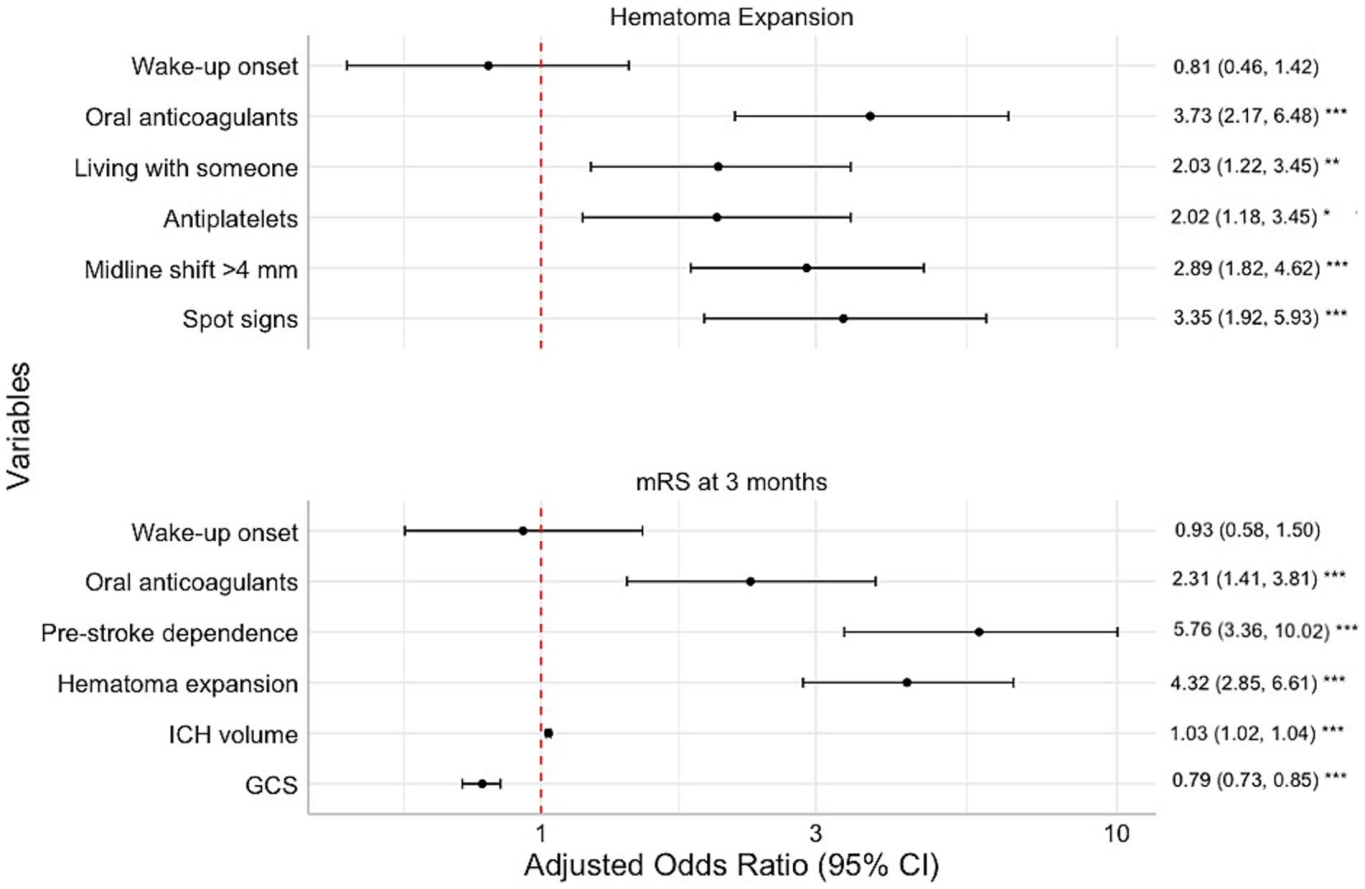
Multivariate analysis of hematoma expansion and modified Rankin Scale scores at 3 months. Increased odds ratio of hematoma expansion and worse functional outcome to the right. Adjusted odds ratio: adjusted common odds ratio for higher mRS at 3 months. GCS, Glasgow Coma Scale; ICH, intracerebral hemorrhage. ***p < 0.001, **p < 0.01, *p < 0.05.

In multivariable analysis, OACs, antiplatelets, midline shift >4 mm, and living with someone were independently associated with HE, while WU onset was not (Figure 2). For alternative HE definitions, antiplatelets did not reach significance when HE was defined as ICH volume growth >6 mL or >33%. When HE was defined as ICH volume growth >12.5 mL or 33%, only OACs and living with someone remained significant. Baseline CTA was available in 309/433 (71%) patients with a follow-up CT, and this group with data on both imaging modalities was subjected to a secondary multivariable analysis. Here, CTA spot signs were independently associated with HE, along with OACs, midline shift >4 mm, and living with someone. NIHSS scores were available in 338/433 (78%) patients, and a sensitivity analysis including this variable showed similar associated variables as the first multivariable analysis without CTA data. Additional variables initially included in the analysis were atrial fibrillation, black hole sign, blend sign, ICH density, ICH shape, PCC treatment, previous ischemic stroke, and dementia. PCC treatment and atrial fibrillation were excluded due to collinearity issues, while the other variables did not demonstrate statistical significance.

**Figure 3:**
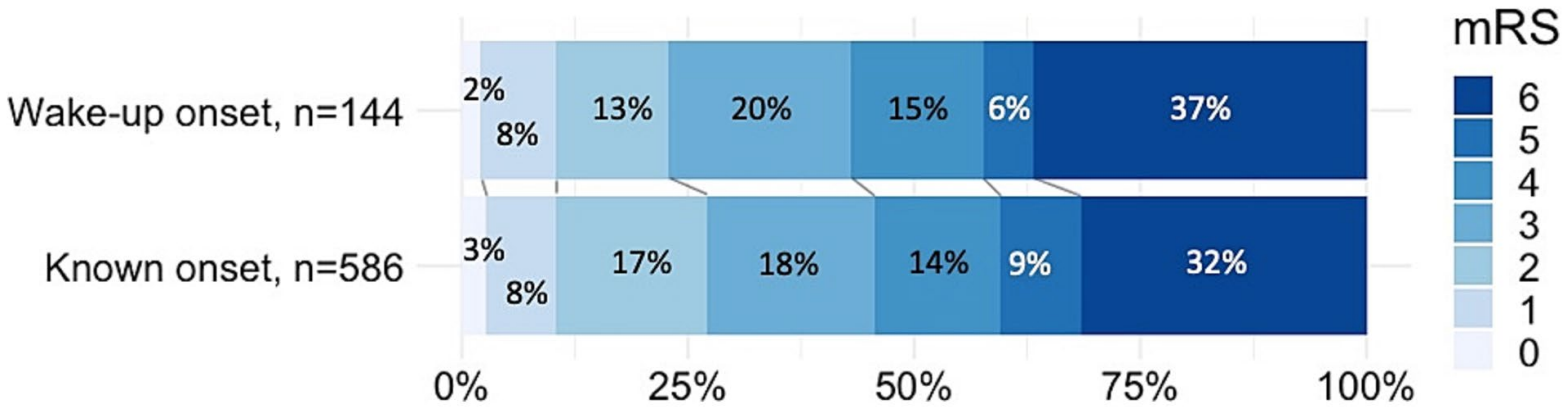
Functional outcomes at 3 months. mRS: modified Rankin Scale.

At 3 months, the mRS score distribution was similar between WU-ICH and known-onset ICH, *p* = 0.35 (Figure 3). Mortality at 3 months was 53/144 (36.8%) in WU-ICH vs. 185/586 (31.6%) in known-onset ICH, *p* = 0.23. In ordinal multivariable analysis, pre-stroke dependence, HE, OACs, baseline ICH volume, and lower admission GCS were associated with higher mRS scores, while WU onset was not (Figure 2). Further variables that were included initially were ICH density, ICH shape, history of antihypertensive medication, atrial fibrillation, and targeted blood pressure treatment. The following variables failed the proportional odds assumption: age, infratentorial ICH, dementia, antiplatelets, systolic blood pressure, and atrial fibrillation. The other variables did not reach significance.

## 4 Discussion

Our results show that patients with wake-up ICH have a similar risk of HE, functional outcomes, and death as those with known-onset ICH. While similar in age, sex, symptom severity, and baseline hematoma volumes, the groups differed on a few pre-stroke characteristics, with dementia, pre-stroke dependence, and the use of oral anticoagulants being more common in WU-ICH. In multivariable analyses, all variables independently associated with HE or worse outcomes have been previously reported (5, 14, 15).

We found only one other previous study investigating HE in WU-ICH, with similar results. One study reported no difference of HE in WU-ICH compared to known-onset ICH (32.4% vs. 24%, *p* = 0.3) (3). It has, however, been reported that HE is more common in patients admitted early after symptom onset (16, 17). In our study, HE occurred in 31.2% of WU-ICH patients despite a median LKW to CT time of 10.3 h, compared to 34.6% in known-onset ICH, with a median onset to CT time of 1.8 h. If ICH onset occurred early during the asleep period, we would have expected HE to be less common in WU-ICH. It has been reported that known-onset ICH peaked between 6:00–8:00 and 16:00–20:00 (2). Blood pressure surges in the early morning have previously been linked to an increased risk of stroke (18). This might influence the onset of ICH similarly in patients who are asleep, meaning that the true onset of WU-ICH could be close to awakening. If that is the case, treatments targeting HE could benefit patients with WU-ICH arriving soon after waking up.

Among studies of acute therapies aimed at reducing HE, the proportion of patients with HE was 22% in ATACH-II, 34% in INTERACT-2, 27% in TICH-2, and 24% in ANNEXA-4 (19–22). The frequency of HE in our study is in the upper range compared to previous reports, and the frequency of HE in WU-ICH at 31% is similar to HE in studies that only included patients within a narrow time window after onset (19–21).

Our study showed no significant differences in functional outcomes or mortality between WU-ICH and known-onset ICH. This is in line with findings from two previous studies, although another study reported higher 1-month mortality for WU-ICH (1, 3, 4). Our findings of a higher prevalence of pre-stroke dependence in WU-ICH are consistent with previous publications (1, 3). The proportion of patients with WU-ICH was 12.5% in our study, compared to 13.5, 14.7, and 20.3% in the above-mentioned studies (1, 3, 4). While pre-stroke dependence and the use of OACs were more common in WU-ICH and independently associated with poor functional outcome, we found that poor functional outcome was not significantly more common in WU-ICH. It is possible that patients with WU-ICH represent a heterogeneous group, including those with onset shortly before awakening and those with onset earlier during sleep.

Our study has limitations requiring consideration. As this study was observational and retrospective, not all patients had a baseline CTA and a follow-up CT, which restricts the robustness of the results. The proportion of patients without a follow-up CT did not differ between WU-ICH and known-onset ICH. In a sensitivity analysis comparing cases with and without a follow-up CT, we found higher mortality but also more frequent mRS 0–1 in patients without a follow-up CT, and no difference in baseline ICH volumes. It is possible that patients with very severe ICH were not re-imaged due to early death or being deemed to be in a terminal state, while clinically stable or improving patients with milder symptoms and small ICH also do not always undergo CT follow-up. Similar proportions of patients without follow-up CT have been reported in other retrospective studies, one of which reported both a worse prognosis and a larger proportion of patients without neurological deficits (23, 24). Another study reported that patients without follow-up CT had a larger baseline ICH volume and worse outcomes (25). The time between initial CT and follow-up CT was longer in WU-ICH, which could also affect the HE analysis. Furthermore, 92/763 (12%) of cases in our study underwent neurosurgery before any follow-up CT was performed, which eliminated them from the analysis of HE. A sensitivity analysis of early neurological deterioration or death <72 h showed no association with wake-up onset (Supplementary material). While early neurological deterioration and early death can be caused by HE, not all patients with HE deteriorated within 72 h and conversely some patients with a stable ICH volume had early neurological deterioration. NIHSS scores were only documented in 509/763 (67%) patients, which may be partially explained by the clinical observation that NIHSS assessment may sometimes be omitted in unconscious patients. While these limitations may have influenced our findings, the primary outcome aligns with the only previously published study on HE in WU-ICH (3).

## 5 Conclusion

Wake-up ICH had a similar risk of HE, neurological deterioration, and similar 3-month outcomes compared to known-onset ICH. If HE is similarly common in WU-ICH as in known-onset ICH, excluding patients with WU-ICH from future trials targeting HE may be unwarranted.

## Data Availability

Anonymized data can be made available by the authors upon reasonable request from a qualified researcher.
