# Supplementary material for: Wake-up intracerebral hemorrhage: hematoma expansion and outcomes
Source: Front Neurol. 2025 Jul 25;16:1620170. doi: 10.3389/fneur.2025.1620170 (PMC12331591; doi:10.3389/fneur.2025.1620170)
Supplement: Supplementary file 1 [file Table_1.DOCX]

Supplementary Material

# Supplementary Data

## Methods

### Sensitivity analysis

We performed a multivariable regression analysis of the composite endpoint of early neurological deterioration or death <72 hours. Pre-determined variables were ICH volume at baseline, systolic blood pressure, infratentorial ICH, GCS, antithrombotic use, and hematoma expansion. Wake-up onset was also included in the regression model and kept throughout the analysis. Further variables were added if they differed on baseline with a p-value <0.10. Variance inflation factor was used to evaluate collinearity. Non-significant values (p>0.05) were removed from the model. For variables with >10 % missing values, we considered whether the variables were missing at random. For variables missing not at random, we performed sensitivity analyses.

## Results

All patients (763) had available data on early neurological deterioration or death <72 hours. The composite endpoint was found in 40/147 (27.2%) in WU-ICH and 180/616 (29.2%) in known onset ICH, p=0.63. It was more common in patients with hematoma expansion, 79/147 (53.7%) versus 69/286 (24.1%), p<0.001. The following variables were included in the multivariable analysis due to baseline differences (Supplementary table 1): Age, pre-stroke dependence, previous ischemic stroke, history of hypertension, atrial fibrillation, dementia, baseline NIHSS, last known well to CT time, midline shift, the black hole sign, the blend sign, fluid level, ICH shape score, intraventricular extension, and hydrocephalus. Spot signs were included in a separate analysis of patients undergoing CTA at admission. Baseline NIHSS was missing in 254/763 (33%). Atrial fibrillation was removed due to collinearity issues with OAC treatment. Remaining significant variables are reported in Supplementary table 2. Wake-up onset was not significantly associated with early neurological deterioration or death <72. Exploratory analyses, including hematoma expansion, spot signs, and NIHSS, respectively did not alter the results.

# Supplementary Figures and Tables

Supplementary table 1. Baseline differences

|  | Neurological deterioration or death, n=220 | No neurological deterioration or death, n=543 |  |
| --- | --- | --- | --- |
|  | n (% ) or median (IQR) | n (% ) or median (IQR) | p |
| Age | 73 (62-81) | 66 (54-77) | <0.001 |
| Sex (f) | 90 (40.9%) | 223 (41.1%) | 0.97 |
| Pre-stroke dependence | 48 (21.8%) | 80 (14.8%) | 0.02 |
| Living alone | 63 (29.3%) | 144 (26.9%) | 0.51 |
| Medical history |  |  |  |
| Previous ICH | 16 (7.3%) | 31 (5.7%) | 0.42 |
| Previous IS | 28 (12.7%) | 46 (8.5%) | 0.08 |
| Hypertension | 136 (61.8%) | 286 (53.0%) | 0.03 |
| Diabetes | 32 (14.5%) | 74 (13.7%) | 0.75 |
| Atrial fibrillation | 55 (25.0%) | 85 (15.7%) | 0.003 |
| Dementia or cognitive impairment | 26 (11.8%) | 28 (5.2%) | 0.001 |
| Medications at onset |  |  |  |
| Oral anticoagulant | 57 (25.9%) | 79 (14.5%) | <0.001 |
| Antiplatelet | 51 (23.2%) | 96 (17.7%) | 0.08 |
| Antihypertensive | 122 (55.5%) | 260 (48.0%) | 0.06 |
| At admission |  |  |  |
| NIHSS | 15 (11-19) | 11 (4-18) | <0.001 |
| GCS | 14 (11-15) | 14 (9-15) | 0.25 |
| Systolic blood pressure | 180 (155-200) | 170 (147-200) | 0.008 |
| ICH score | 2 (1-2) | 1 (0-2) | 0.02 |
| Medical treatments |  |  |  |
| PCC | 37 (17.1%) | 69 (12.8%) | 0.13 |
| Vitamin K | 21 (9.7%) | 47 (8.7%) | 0.68 |
| Targeted blood pressure treatment | 164 (75.6%) | 386 (71.3%) | 0.24 |
| Time from LKW to first CT | 1.9 h (1.3-3.7) | 2.25 h (1.5-6.4) | 0.001 |
| First CT scan |  |  |  |
| ICH volume (ml) | 20.0 (7.3- 44.3) | 11.4 (4.0 - 28.9) | <0.001 |
| IVH | 118 (53.6%) | 233 (43.0%) | 0.008 |
| Hydrocephalus | 99 (45.0%) | 188 (34.6%) | 0.007 |
| Midline shift (>4 mm) | 93 (42.3%) | 150 (27.7%) | <0.001 |
| Blend sign | 31 (14.2%) | 46 (8.7%) | 0.03 |
| Black hole sign | 71 (32.4%) | 135 (25.5%) | 0.05 |
| Fluid sign | 10 (4.6%) | 9 (1.7%) | 0.02 |
| Barras scale density | 1 (1-2) | 1 (1-2) | 0.16 |
| Barras scale shape | 2 (1-3) | 1 (1-2) | <0.001 |
| CTA on arrival | 138 (62.7%) | 359 (66.1%) | 0.37 |
| Spot sign(s) | 76 (55.1%) | 115 (32.0%) | <0.001 |
| Hematoma expansion | 79 (53.4%) | 68 (23.9%) | <0.001 |

CT: Computed Tomography; CTA: Computed Tomography Angiography; GCS: Glasgow Coma Scale; NIHSS: NIH stroke scale; ICH: Intracerebral hemorrhage; IVH: Intraventricular Hemorrhage; IS: Ischemic stroke; LKW: Last Know Well; PCC: Prothrombin complex concentrate.

Supplementary table 2. Multivariable regression analysis of early neurological deterioration or death <72 hours after ICH onset.

| Variable | Adjusted Odds Ratio (95% CI) |
| --- | --- |
| Wake-up onset | 0.87 (0.57,1.33) |
| Hematoma expansion | 3.90 *** (2.51,6.09) |
| Oral anticoagulants | 2.52 *** (1.67,3.79) |
| Spot signs | 2.30 *** (1.48,3.59) |
| Midline shift | 2.17 *** (1.46,3.21) |
| Barras shape score | 1.35 *** (1.16,1.57) |
| Baseline GCS | 1.11 *** (1.06,1.17) |

Adjusted odds ratios för hematoma expansion and spot signs are obtained from sensitivity analyses. *** p < 0.001; ** p < 0.01; * p < 0.05. GCS: Glasgow Coma Scale.
